# Antibiotic exposure and risk of weight gain and obesity: protocol for a systematic review

**DOI:** 10.1186/s13643-017-0565-9

**Published:** 2017-08-24

**Authors:** Heidi Dutton, Mary-Anne Doyle, C. Arianne Buchan, Shuhiba Mohammad, Kristi B. Adamo, Risa Shorr, Dean A. Fergusson

**Affiliations:** 1 Division of Endocrinology & Metabolism, University of Ottawa, Ottawa, ON Canada; 2 Ottawa Hospital Research Institute, Ottawa, ON Canada; 3 School of Epidemiology, Public Health and Preventive Medicine, Faculty of Medicine, University of Ottawa, Ottawa, ON Canada; 4 The Ottawa Hospital, 1967 Riverside Drive, Riverside Campus, 4th floor, Ottawa, ON K1H 7W9 Canada; 5 Division of Infectious Diseases, University of Ottawa, Ottawa, ON Canada; 6 School of Human Kinetics, Faculty of Health Sciences, University of Ottawa, Ottawa, ON Canada; 7 Department of Pediatrics, Faculty of Medicine, University of Ottawa, Ottawa, Canada

**Keywords:** Antibiotics, Weight gain, Obesity, Gut, Microbiome, Systematic review

## Abstract

**Background:**

The prevalence of obesity is increasing worldwide, and there is growing interest in better delineating the role of the human gut microbiome in this phenomenon. Obesity-specific gut microbiome features have been observed in both human and animal studies, and these variations appear to play a causative role in increasing body weight. There is evidence that antibiotics can modify the composition and diversity of the gut microbiome and that this may contribute to body weight changes. The primary objective of the proposed systematic review is to evaluate and synthesize the existing evidence evaluating the possible association between antibiotic use, weight gain, and obesity.

**Methods:**

A comprehensive search of the MEDLINE and EMBASE databases will be performed. Both randomized and non-randomized studies (excluding case reports) in neonates, children, adults, and pregnant women will be included. The exposure of interest is antibiotics of any type, duration, and route given for any indication. All included studies must have a comparator group. The primary outcomes are the development of overweight and obesity. Secondary outcomes are percent weight-change from baseline and change in body mass index or waist circumference. Additional secondary outcomes in pregnant women are gestational weight gain, postpartum weight retention, offspring birth weight, childhood weight, and obesity. Risk of bias of included trials will be performed. Two reviewers will screen and perform data extraction independently.

**Discussion:**

This systematic review will summarize the existing evidence evaluating the association between antibiotic use, weight gain, and obesity and facilitate the identification of important gaps and uncertainties in the literature.

**Systematic review registration:**

PROSPERO CRD42017069177

**Electronic supplementary material:**

The online version of this article (doi:10.1186/s13643-017-0565-9) contains supplementary material, which is available to authorized users.

## Background

Obesity is increasing in prevalence worldwide [1] and is now considered by many organizations to be a chronic disease [2, 3]. Numerous factors are felt to have contributed to the increased prevalence of this condition, including increased access to palatable high-calorie foods, decreasing rates of physical activity, increasingly sedentary lifestyles, and use of medications that are known to be associated with weight gain [4]. In recent years, interest in the role of the human intestinal microbiome in various chronic conditions has been rapidly increasing [5]. The human gut contains between ten trillion and 100 trillion microorganisms, with the two most abundant bacterial phyla being the *Bacteroidetes* and the *Firmicutes*. The microbiome composition varies with many factors including sex, age, diet, and ethnicity [6]. In both animal and human studies, obesity has been found to be associated with a lower proportion of *Bacteroidetes* to *Firmicutes* [7, 8], and a relative increase in *Bacteroidetes* has been described with weight loss [8]. Obesity is also associated with decreased gut microbial diversity compared to lean individuals [9]. In pediatric populations, higher numbers of bifidobacteria at age 6 and 12 months have been shown to be predictive of childhood overweight and obesity at 7 years of age [10].

Evidence that these variations in gut bacteria profiles likely play a causal role in weight gain has been elucidated through fecal transplantation studies. For example, adult germ-free C57BL/6J mice colonized with microbiota harvested from obese (*ob/ob*) mice gained a higher percentage of body fat over 2 weeks compared to those colonized with microbiota from lean (+/+) mice donors [11]. Similarly, Ridaura et al. studied human twins discordant for obesity and found that fecal microbiota from obese twins transplanted into C57BL/6J germ-free mice resulted in greater increases in adiposity as compared to those mice who received fecal microbiota from lean twins [12]. It has also been shown that adult C57BL/6J germ-free mice gain less weight with a high fat, sugar-rich diet as compared to conventionally raised mice with an intact gut microbiome, further confirming that gut bacteria likely play a role in metabolism [13]. It has been proposed that the variations in gut bacteria associated with obesity may promote increased energy extraction from food passing through the gut via increased production of short-chain fatty acids [14]. Furthermore, the metabolism of bile acids, and certain phospholipids and amino acids, have also been described as factors that may mediate interactions between the microbiota and human metabolism [14].

The administration of antibiotics has been shown to influence the composition of the human gut microbiome [15–18]. For example, Jakobsson et al. found that administration of clarithromycin and metronidazole for treatment of *helicobacter pylori* infection in six individuals resulted in shifts in gut microbial communities and that alterations in gut microbiota composition persisted for up to 4 years post treatment in some participants [17]. Not surprisingly, different classes of antibiotics have been associated with distinctive alterations in the gut microbiota [16]. Given this likely influence of antibiotics on the composition of the gut microbiome, it has been suggested that the use of antibiotic therapies in humans may be contributing to the increasing prevalence of obesity [19, 20].

Previous narrative reviews and opinion papers have cited observational studies in neonatal and pediatric populations suggesting evidence of an association between antibiotic use and increased body weight [21–23]. In adults, this association is based on evidence from very specific disease states, such as endocarditis, *h. pylori* infection, and cystic fibrosis [21]. Evidence of a causal relationship between antibiotic use and weight gain in animal studies is potentially supportive of a similar relationship in humans [19, 23]. However, to our knowledge, no existing reviews have systematically evaluated the evidence that antibiotics may contribute to weight gain in humans. Thus, it remains unclear whether important evidence pertaining to other populations and disease states is being overlooked. Furthermore, no systematic evaluation of the quality of the existing evidence has been undertaken. A systematic evaluation and synthesis of evidence is warranted, given the potential public health implications if indeed antibiotic use in humans contributes to weight gain.

## Objective

The primary objective of this systematic review is to summarize the literature evaluating the potential association between antibiotic administration and weight gain and/or obesity. The evidence will be evaluated in neonates, children, adults, and pregnant women. Antibiotics of any type, duration, or route and for any indication will be captured. Our secondary objective is to evaluate changes in gut microbiome composition and diversity associated with antibiotic-related changes in weight. With this review, we also plan to evaluate gaps or uncertainties in the existing literature and characterize how this may inform future research initiatives.

## Methods/design

This systematic review was designed in accordance with the Preferred Reporting Items for Systematic Review and Meta-Analyses (PRISMA) guidelines [24], with use of a PRISMA-P checklist [25] (see Additional file 1 for completed checklist). The protocol has been registered on PROSPERO (#CRD42017069177).

### Eligibility criteria

#### Population

The populations of interest include adults, pregnant women, children, and infants.

#### Exposure

The intervention of interest is therapeutic use of antibiotics of any dose, class/type, duration, or route given for any indication. It is expected that evidence may pertain to a large variety of disease states and indications. We feel that any restrictions based on antibiotic dose, type, duration, or indication could potentially exclude important evidence, and thus, the exposure eligibility criteria are broad. However, environmental studies, for example evaluating levels of antibiotic metabolites in biologic fluids and whether this is associated with higher body weight, will not be included.

#### Comparators

All included studies must include a control group. This can be either a group that did not receive antibiotics or a group who received a different antibiotic or combination of antibiotics.

#### Outcomes

The outcomes of interest will differ depending on the population subtype. In all populations, the primary outcomes are the development of overweight or obesity. Secondary outcomes are the percent weight-change from baseline and change in body mass index (BMI) or waist circumference. In pregnant women, secondary outcomes will also include maternal outcomes of gestational weight gain and postpartum weight retention and/or fetal outcomes of offspring birth weight and offspring childhood weight or risk of obesity. A final secondary outcome of interest is change in microbiome composition (i.e., change in amount of particular type(s) of bacteria or bacterial diversity from baseline). In order to be eligible for inclusion, studies must report on at least one of the primary or secondary weight-related outcomes.

#### Study designs

Eligible human studies include comparator-controlled, interventional, and observational studies such as case-control, cross-sectional, and prospective or retrospective cohort studies.

### Search strategy

An information specialist who is experienced in the conduct of systematic reviews (RS) developed the initial search strategy with input from one of the reviewers (HD) (see Additional file 2). The search strategy represents a comprehensive electronic search of Medline (Ovid Medline Epub Ahead of Print, In-Process & Other Non-Indexed Citations, Ovid Medline Daily and Ovid Medline 1946–Mar. 2, 2017) and Embase (Embase Classic and Embase 1947–Mar. 2, 2017). Reference lists of included studies and any applicable review studies will also be searched to ensure that further studies meeting eligibility criteria have not been missed by the initial search strategy.

Due to resource and time limitations, only English studies will be included. The anticipated volume of search results is large, and thus, case reports, abstracts, and conference proceedings will be excluded. Review articles will also be excluded. Letters will be evaluated for inclusion on a case-by-case basis. If two reviewers independently feel a letter contains sufficient relevant information, it will be included.

### Study selection

All references retrieved through the search strategy will first be screened based on title and abstract using CrowdScreenSR, a web-based tool. Duplicate references will first be removed, and then, two independent reviewers will independently screen all titles and abstracts against the eligibility criteria (HD with one of MAD, SM, or CAB). Any disagreements will be resolved by a third reviewer. Full text will then be accessed for studies deemed potentially eligible based on title and abstract. Two investigators (HD and one of SM or CAB) will then independently screen the full-text studies to determine eligibility. Disagreements will be resolved by discussion or a third reviewer if necessary. Reasons for study exclusion will be documented.

### Data extraction

Data will be extracted from each study deemed eligible for inclusion with a data extraction tool designed for this purpose using REDCap, a web-based tool. Three reviewers (HD, MAD, and CAB) will independently pilot the human study tool on two studies. The data extraction tools will then be revised based on feedback from the reviewers. Once the tools have been finalized, two reviewers will independently extract relevant data from all eligible studies (HD will extract from all studies, and studies will be divided between MAD, SM, and CAB). Disagreements will be resolved by discussion or, if necessary, by a third reviewer.

For each included study, relevant publication details (author, year published, country), study details (study design, sample size), participant demographics, sample size of exposed and unexposed groups, exposure details (antibiotic class/type, dose, duration, route), and comparator or control group demographics will be extracted. Extracted participant demographics for intervention and control groups will include baseline weight and/or BMI (if applicable), age at antibiotic administration (mean or median), percentage of male sex, diet, smoking, physical activity measures, and ethnicity. For neonatal and pediatric studies, mean birth weight, delivery mode, gestational age, initiation/duration of breastfeeding, and maternal factors (smoking, pregnancy complications, parity, age, gestational weight gain, pre-pregnancy BMI, parity, and indicators of socioeconomic status) will also be extracted. The indication for antibiotic therapy will be extracted, as well as any demographic data relevant to that indication.

The outcomes specified in the eligibility criteria will be extracted for each study, along with the timing of outcome measure in relation to antibiotic exposure. In addition, standard deviation, standard error, and *p* values for all outcomes of interest will be extracted. For all studies, data on changes in microbiome composition following antibiotic administration will be extracted, including changes in particular types of bacteria (e.g., changes in proportion of amount of phyla, class, family, or species), changes in diversity of bacteria, and timing of microbiome evaluation after antibiotic administration.

### Risk of bias assessment

All risk of bias assessments will be performed independently by two reviewers (HD and either MAD, CAB, or SM), with disagreement resolved by discussion or a third reviewer if necessary. Any included interventional study will be assessed using the Cochrane Risk of Bias Assessment Tool [26]. There is no consensus regarding the optimal method of assessing risk of bias in nonrandomized studies [27]. In the proposed review, nonrandomized studies will be evaluated using the Newcastle-Ottawa scale for assessing the quality of nonrandomized studies [28], as our group has previous experience using this tool in the conduct of systematic reviews.

### Results and data synthesis

The results of the systematic review will be synthesized descriptively in terms of basic characteristics of each included study, such as type of study, study population, and year of publication. The primary outcomes will be presented in tabular format, divided into adult, pediatric, and perinatal/fetal evidence. Descriptive results will also be grouped based on characteristics of participants (e.g., age, sex, indication for antibiotics), and antibiotic exposure (e.g., antibiotic class, duration, type of bacterial coverage) will be explored to further evaluate and understand the existing evidence. We will also synthesize any reported changes in gut microbiome and diversity as they relate to our weight-related outcomes. In order to fully assess for gaps in evidence, results will also be evaluated in terms of individual study quality and risk of bias. This will be summarized in a narrative fashion with no formal assessment of evidence strength.

Interventional and observational studies will be summarized separately. We expect that the majority of studies meeting our eligibility criteria will be observational in nature, and we also anticipate significant clinical and methodological heterogeneity between studies. There are a number of potential factors that could confound the relationship between antibiotic use and weight gain, and thus, the adjusted risk estimates will be of greatest interest in the included observational studies. It is also likely that studies will adjust for varying combinations of potential confounders.

For interventional studies reporting the same outcome (e.g., risk of obesity), we will pool dichotomous data using random effects models and reporting relative risks and 95% confidence intervals. For continuous data, for example, change in weight or BMI, we will pool data using random effects models and reporting either mean differences (if possible) or standardized mean differences (if necessary due to different measurement scales) and 95% confidence intervals. Specifically, mean between group change score differences (or standardized mean differences) will be assessed. As a sensitivity analysis, we will also analyze between group final values. In order to assess heterogeneity, we will first consider factors such as study population, type of antibiotic and indication, comparator group, and means of assessing study outcome. Comparison of these factors between studies will allow for an assessment of clinical heterogeneity. Next, the *I* ^2^ statistic will be calculated to assess statistical heterogeneity, using a cutoff of ≥ 0.75 to define significant heterogeneity between studies [29]. If the *I* ^2^ value is below the pre-specified cutoff, and clinical heterogeneity is also deemed to be reasonably low, meta-analysis will be undertaken.

### Reporting of the review

The methods and results of the systematic review will be reported in accordance with the PRISMA guidelines [25].

## Discussion

In our systematic review, we will synthesize the evidence evaluating the association between antibiotic use and weight gain and risk of obesity in humans. It is expected that there will be significant heterogeneity between the studies included, particularly in terms of study design, study population, antibiotic type and indication, and conditions being treated. We anticipate that we may have difficulty drawing generalized conclusions regarding the possible effects of antibiotics on weight gain. However, given the potential public health implications of such an association, it is important to summarize the existing evidence in order to facilitate the identification of important gaps and uncertainties in the literature. Through such a summary, important hypotheses regarding the effect of antibiotics on body weight and adiposity can be generated. This review will provide a solid foundation for the design and implementation of future studies that can better clarify the relationship between antibiotic use and weight gain in humans.

## Additional files

Additional file 1: PRISMA-P 2015 Checklist (.pdf)—PRISMA-P checklist for study protocol. (PDF 163 kb)

Additional file 2: Search Strategy (.pdf)—preliminary search strategy. (PDF 132 kb)

## Abbreviations

BMI: Body mass index
PRISMA: Preferred Reporting Items for Systematic Review and Meta-Analyses
